# Metabolic Modeling of Streptococcus mutans Reveals Complex Nutrient Requirements of an Oral Pathogen

**DOI:** 10.1128/mSystems.00529-19

**Published:** 2019-10-29

**Authors:** Kenan Jijakli, Paul A. Jensen

**Affiliations:** a Department of Bioengineering, University of Illinois at Urbana-Champaign, Urbana, Illinois, USA; b Carl R. Woese Institute for Genomic Biology, University of Illinois at Urbana-Champaign, Urbana, Illinois, USA; c Department of Microbiology, University of Illinois at Urbana-Champaign, Urbana, Illinois, USA; University of California, Irvine

**Keywords:** *Streptococcus mutans*, dental caries, flux balance analysis, metabolic modeling

## Abstract

Streptococcus mutans is a Gram-positive bacterium that thrives under acidic conditions and is a primary cause of tooth decay (dental caries). To better understand the metabolism of S. mutans on a systematic level, we manually constructed a genome-scale metabolic model of the S. mutans type strain UA159. The model, called iSMU, contains 675 reactions involving 429 metabolites and the products of 493 genes. We validated iSMU by comparing simulations with growth experiments in defined medium. The model simulations matched experimental results for 17 of 18 carbon source utilization assays and 47 of 49 nutrient depletion assays. We also simulated the effects of single gene deletions. The model’s predictions agreed with 78.1% and 84.4% of the gene essentiality predictions from two experimental data sets. Our manually curated model is more accurate than S. mutans models generated from automated reconstruction pipelines and more complete than other manually curated models. We used iSMU to generate hypotheses about the S. mutans metabolic network. Subsequent genetic experiments confirmed that (i) S. mutans catabolizes sorbitol via a sorbitol-6-phosphate 2-dehydrogenase (SMU_308) and (ii) the Leloir pathway is required for growth on complex carbohydrates such as raffinose. We believe the iSMU model is an important resource for understanding the metabolism of S. mutans and guiding future experiments.

**IMPORTANCE** Tooth decay is the most prevalent chronic disease in the United States. Decay is caused by the bacterium Streptococcus mutans, an oral pathogen that ferments sugars into tooth-destroying lactic acid. We constructed a complete metabolic model of S. mutans to systematically investigate how the bacterium grows. The model provides a valuable resource for understanding and targeting S. mutans’ ability to outcompete other species in the oral microbiome.

## Abstract (precis)

Tooth decay is the most prevalent chronic disease in the United States. Decay is caused by the bacterium Streptococcus mutans, an oral pathogen that ferments sugars into tooth-destroying lactic acid. We constructed a complete metabolic model of S. mutans to systematically investigate how the bacterium grows. The model provides a valuable resource for understanding and targeting S. mutans’ ability to outcompete other species in the oral microbiome.

## INTRODUCTION

Streptococcus mutans is one of over 600 species of bacteria in the oral microbiome (1). This Gram-positive, lactic acid bacterium thrives in the oral environment in part due to its metabolic flexibility. S. mutans can feed on several carbohydrates (2) and has complex, interdependent amino acid auxotrophies (3). S. mutans is the primary cause of tooth decay (dental caries). By fermenting a wide array of dietary sugars into lactic acid, S. mutans creates a highly acidic microenvironment near the tooth surface (as low as pH 3.0) (4). The lactic acid demineralizes the tooth structure, resulting in decay.

Understanding the acidogenic capabilities of S. mutans requires an unbiased, systems-level approach. Previous studies have shown that acid production and tolerance in S. mutans require large changes in gene expression and metabolic pathway utilization (5). For example, decreasing pH increases glycolytic activity and branched-chain amino acid synthesis without increasing cell growth (6). A drop in pH is also accompanied by an increased expression of F-ATPases to maintain a higher intracellular pH (7).

Mathematical models aid in our understanding of how an organism’s genes collectively give rise to a phenotype. Models translate bioinformatic features (differential expression, presence/absence of genes) into biological function (flux distributions, uptake and secretion rates, and fitness). Constraint-based reconstruction and analysis (COBRA) of genome-scale models is widely used to integrate genetic and metabolic data to produce phenotypic predictions (8, 9). Models of microbial metabolism and transcriptional regulation predict responses to gene deletions (10, 11), mutation (12, 13), metabolic shifts (14, 15), and long-term evolution (16, 17). The models identify emergent properties of a metabolic network, including links between pathways and interdependencies among genes (18, 19).

We present iSMU v1.0, a genome-scale metabolic model for the S. mutans type strain UA159. Our model is manually curated using multiple databases, literature evidence, and phenotyping experiments. Model predictions were tested using growth and mutagenesis experiments, demonstrating the utility of the iSMU model in guiding predictions and experiments. Our investigation of S. mutans focused on metabolism for two reasons: (i) the primary metabolic products of S. mutans, lactic acid and biofilm matrix, are responsible for the pathogen’s cariogenicity, and (ii) metabolic networks are among the best-characterized intracellular networks with established computational techniques. Given metabolism’s central role in cariogenesis, we believe the iSMU model will improve our understanding of S. mutans’ role in oral health.

## RESULTS

### Manual curation produces an annotated metabolic model for S. mutans.

**FIG 1:**
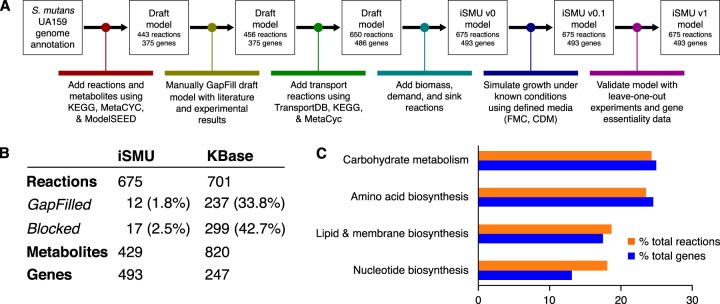
(A) Reconstruction of the S. mutans metabolic network began with an annotated UA159 genome. The draft model was refined with bioinformatics databases and experimental results. (B) The manually reconstructed model (iSMU) has fewer GapFilled (non-gene-associated) and blocked reactions than a model generated automatically by the KBase system. Metabolite counts do not reflect compartmentalization, e.g., “glucose[c]” and “glucose[e]” are counted as one metabolite. (C) The reactions and genes in the iSMU model are distributed across a range of KEGG subsystems.

**FIG 2:**
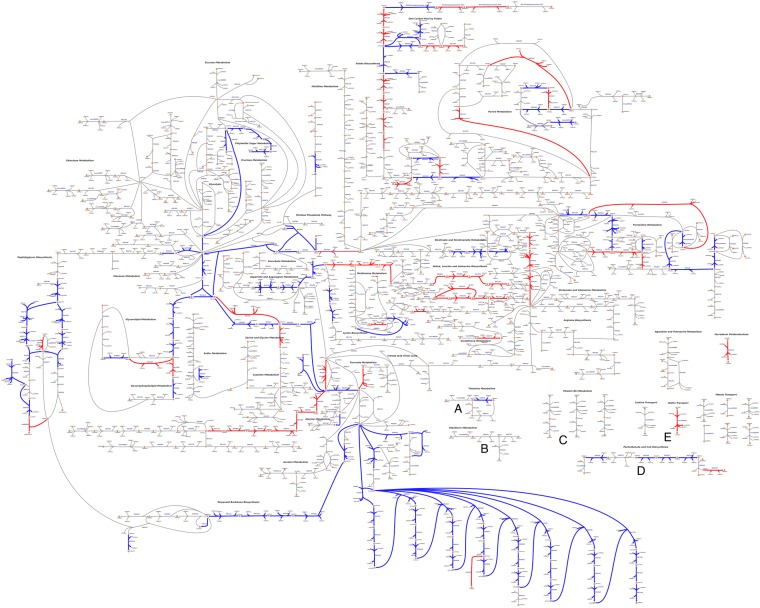
A custom pathway map shows all reactions in the iSMU model. (A high-resolution image is available as Data Set S4 in the supplemental material.) S. mutans UA159 appears to lack complete pathways for synthesizing thiamine (A), riboflavin (B), pyridoxine (C), pantothenate (D), and biotin (E). Reactions are colored by agreement between the essentiality predictions of the associated genes and Tn-seq data from the work of Shields et al. (31). Blue reactions agree with the Tn-seq essentiality results; red reactions disagree. Overall agreement between the data sets is 84.8% (Fig. 4).

We manually reconstructed an *in silico* metabolic model for S. mutans type strain UA159 (Fig. 1A). Our model, named iSMU for “*in silico* S. mutans,” includes major metabolic pathways for carbohydrate metabolism and synthesis of amino acids, nucleotides, lipids, vitamins, and cofactors. The model includes 675 reactions transforming 429 metabolites. The reactions are catalyzed by the products of 493 genes (Fig. 1B). Figure 2 shows all reactions in the iSMU model.

Our assembly of iSMU began with reaction databases and an annotated genome. Draft models assembled from genome annotations are often incomplete because of gaps in the genome annotation or spontaneous reactions that lack an associated enzyme. Several computational methods attempt to identify and add these missing reactions in a process called GapFilling (20). Rather than rely on automated GapFilling algorithms, we manually GapFilled iSMU by examining the reactions in each pathway and testing for growth in chemically defined medium (CDM) with glucose as the sole carbon source. We attempted to close gaps in any pathway that (i) was complete except for a small number of reactions or (ii) was blocked (unable to carry flux) due to metabolites that could not be produced or consumed. We also attempted to find unannotated or misannotated genes that could catalyze the GapFilled reactions. Compared to other metabolic reconstructions, our manually GapFilled model contains fewer incomplete pathways (Fig. 1B; see also Table S3 in the supplemental material). On average, published metabolic models of well-studied organisms lack gene annotations for 53% of the models’ reactions (Table S3). These models also average 32.7% blocked reactions, i.e., reactions that cannot carry a steady-state flux because they lack upstream or downstream pathways. Our iSMU model has only 23% reactions without an associated enzyme and 2.5% blocked reactions.

Metabolic models simulate growth by collecting cellular building blocks into a biomass reaction. The biomass reaction is used as the objective function for metabolic simulations. A nonzero flux through the biomass reaction indicates growth in the metabolic environment specified by the model’s inputs (called exchange reactions). We modified the biomass reaction from a model of Enterococcus faecalis V583 (21) to create a biomass reaction for S. mutans UA159. Both E. faecalis and S. mutans are lactic acid bacteria, with similar metabolic capabilities. To tailor the biomass reaction to S. mutans, we changed the relative ratios of nucleotides and amino acids. We also changed the cell membrane composition to reflect membrane sugar polysaccharides specific to S. mutans. We replaced UDP-*N*-acetyl-d-galactosamine, which, based on genetic evidence, is not produced by S. mutans, with UDP-*N*-acetyl-d-mannosamine and UDP-*N*-acetyl-d-glucosamine (22). We also adjusted the cell wall fatty acids to their measured proportions at pH 7.0 (23). The final biomass reaction consumes 56 metabolites to produce a unit of biomass.

### Manual curation improves model consistency and accuracy.

Several software systems can generate draft metabolic models using reaction databases and annotated genomes, a process called automatic reconstruction. Typically, metabolic models are reconstructed by first using a software package to generate a draft model and then manually curating that draft model to increase its consistency and reconcile it with other biological data. Our first attempt at reconstructing the metabolism of S. mutans used a draft model from the KBase system (24). Unfortunately, the draft model lacked many of the metabolic features of lactic acid bacteria and included several subsystems known to be inactive in homofermentative anaerobes. We therefore abandoned the KBase model and began a manual reconstruction process. We compared the final iSMU model to the KBase model to quantify the disagreement between the manual and automated reconstruction pipelines.

Our manually curated reconstruction differs substantially from the reconstruction produced automatically by KBase. Our iSMU model has 3.7% fewer reactions than the KBase model (675 versus 701, respectively) but 99.5% more genes (493 versus 247, respectively) (Fig. 1B). Thus, the manually curated model has a larger proportion of gene-associated reactions than the automated reconstruction. The dearth of gene associations in the KBase model is due in part to the 237 reactions added during GapFilling, since GapFilled reactions are added without genomic evidence for the reaction. By comparison, our iSMU model required only 12 GapFilled reactions to enable growth on defined medium.

The KBase model contains 41% more metabolites than our iSMU model. Unfortunately, 22 of the metabolites in the KBase model are “dead-end” metabolites that lack either a producing or a consuming reaction. The dead-end metabolites block flux through 299 (43%) of the KBase model’s reactions. At steady state, these blocked reactions cannot carry flux or be analyzed using flux balance analysis (FBA). About 2.5% of the reactions in our iSMU model are blocked, indicating more complete reaction pathways than in the model created by automated reconstruction.

### A comparison of iSMU to other microbial genome-scale models.

Existing genome-scale metabolic models play an important role in the reconstruction of models for new species. It is common for the new models to be built by comparison to the older ones, just as our iSMU model builds on the biomass reaction from an E. faecalis model. Automated reconstruction pipelines in particular depend on existing models (24–26). The earliest reconstruction of a microbial genome-scale metabolic model was for the Gram-negative Escherichia coli K-12 MG1655 (27), and this model has had periodic updates, culminating in the model iML1515 (28). Among the first Gram-positive reconstructions was a model of Bacillus subtilis 168 (29). The latest B. subtilis 168 model is iBSU1144 (30). Both models represent a well-studied microorganism with a wealth of existing data.

Relative to the total number of genes in each organism, iSMU contains 24.2% of all S. mutans genes, while iML1515 contains 35% of all E. coli genes and iBSU1144 contains 16.5% of all B. subtilis genes. The high fraction of modeled genes in the E. coli model may reflect its 2-decade history of curation and updates. The number of blocked reactions in iSMU is significantly lower than in both other models (2.5% compared to 35.6% in iML1515 and 67.9% in iBSU1144). Finally, the number of components that are required for the production of biomass in iML1515 and iBSU1144 is higher than in iSMU. The biomass reaction of iML1515 includes 101 components, nearly twice the number of components found in iSMU’s biomass reaction (Table S4).

Primary metabolism (metabolism of carbohydrates, amino acids, nucleotides, lipids, fatty acids, and cell membrane components) includes 308 genes in iSMU, or 62.5% of all genes in the model. Primary metabolism in iML1515 includes 607 genes (40%) and in iBSU1144 includes 398 genes (55.4%). The largest proportion of genes in iSMU are involved in carbohydrate metabolism (120 genes, 24.3%). This is followed by amino acid metabolism (118 genes, 23.9%) and then metabolism of lipids, fatty acids, and cell membrane components (84 genes, 17%), and then, finally, nucleotide metabolism (63 genes, 12.7%) (Fig. 1C). In contrast, the plurality of genes in iML1515 and iBSU1144 were involved in amino acid metabolism. In iML1515, amino acid metabolism involved 229 genes (15.1%), followed by carbohydrate metabolism (205 genes, 13.5%); metabolism of lipids, fatty acids, and cell membrane components (179 genes, 11.8%); and finally nucleotide metabolism (131 genes, 8.6%). There were 195 genes involved in amino acid metabolism in iBSU1144 (27.1%), followed by 122 genes involved in carbohydrate metabolism (17%). Metabolism of lipids, fatty acids, and cell membrane components and metabolism of nucleotides were tied at 100 genes each (13.9%). Compared to E. coli or B. subtilis, a larger fraction of the genome of S. mutans is associated with carbohydrate metabolism. The focus on carbohydrates is consistent with S. mutans’ need to scavenge dietary sugars while living solely in the human mouth.

**FIG 3:**
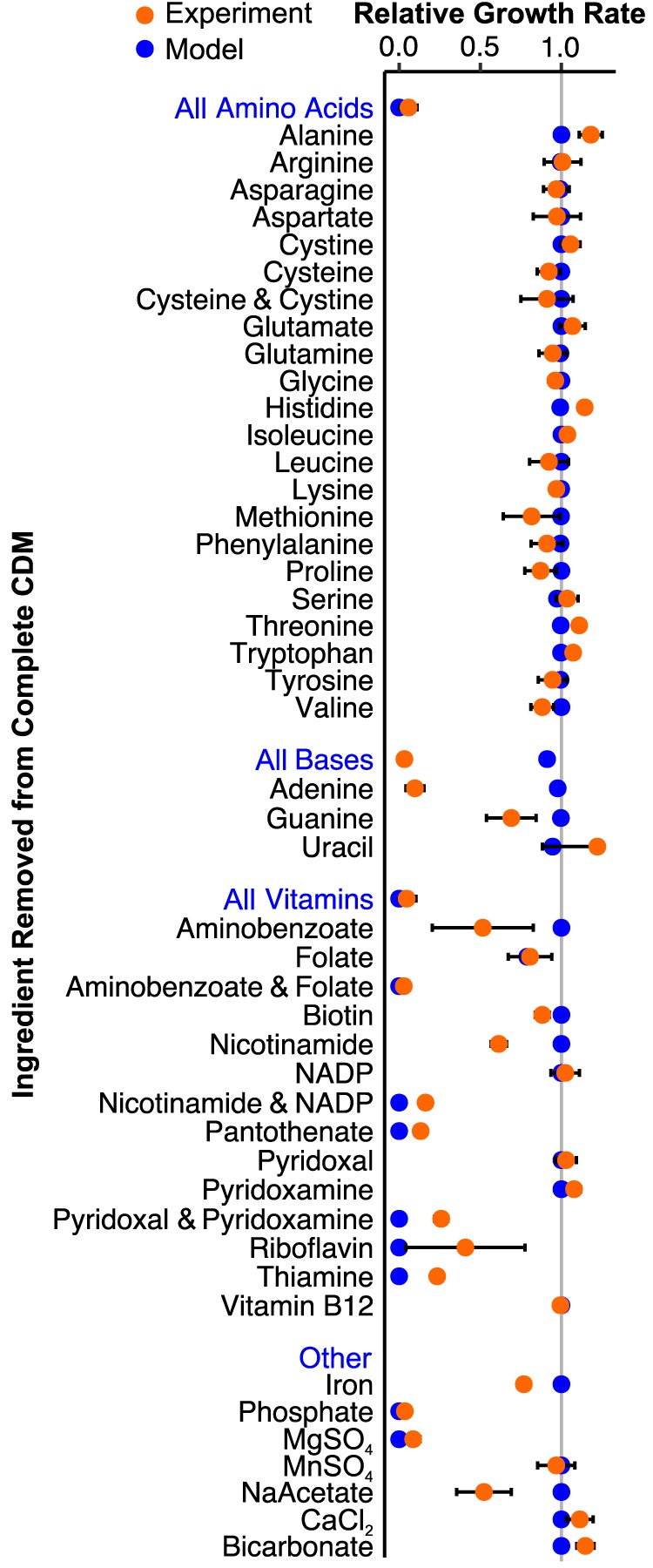
Model predictions (blue) match growth experiments for S. mutans UA159 in defined medium (orange). Growth rate was measured for CDM lacking the specified component(s). Blue labels indicate removal of all components listed below. Experimental data are means from three independent trials with error bars representing the standard deviation. Growth rates are normalized to S. mutans UA159 grown in complete CDM.

Only 54 genes in iSMU are involved in the biosynthesis and metabolism of vitamins and cofactors, compared to 178 genes in iML1515 and 117 genes in iBSU1144. The low number of biosynthetic genes is consistent with S. mutans’ dependence on its host for these nutrients. S. mutans is auxotrophic for several vitamins and cofactors (Fig. 3), whereas the free-living E. coli and B. subtilis can synthesize their own secondary metabolites.

### Gene deletion simulations match experimental data.

Metabolic models contain chemical reactions and gene associations that link reactions to their corresponding enzymes. Gene associations are expressed as logical statements describing the required gene products for a reaction to carry flux. An enzymatic complex of two proteins is expressed using “and” (subunit 1 and subunit 2). A pair of isozymes that could each independently catalyze a reaction would be written with an “or” (isozyme 1 or isozyme 2). Flux balance analysis and the model’s gene associates can be combined to simulate the effects of gene deletions on growth. The logical rules in the gene associations are evaluated to identify reactions that cannot carry flux in a deletion strain. Reactions that cannot carry flux are removed from the model before calculating the maximum biomass flux. Deletion of an essential gene will prevent any nonzero biomass flux. Comparing experimentally determined essential genes with the model’s predictions validates the model’s gene associations.

We simulated the effects of all single deletions for the 493 genes in the iSMU model (Data Set S5). We compared the *in silico* deletions to two experimental gene deletion studies in S. mutans UA159: a transposon mutagenesis sequencing (Tn-seq) experiment (31) and a screen of an ordered array of single-gene deletion strains (32). We checked every disagreement between the model simulations and the experimental data to correct errors in gene associations. Changes were validated by literature searches whenever possible.

**FIG 4:**
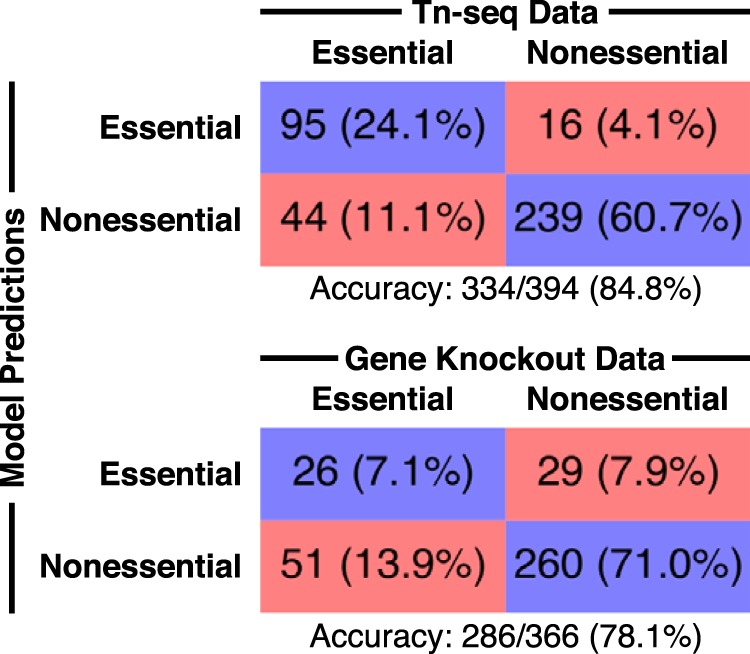
iSMU essentiality predictions align with two experimental studies. Shields et al. (31) (top) used transposon mutagenesis sequencing (Tn-seq) to identify essential genes in the defined medium FMC. Quivey et al. (32) (bottom) screened a library of single-gene knockout strains for growth in rich medium. Blue boxes indicate the number (percentage) of genes in the model and data set that are both essential or both nonessential. Red boxes indicate disagreements between the model and experiments.

The Tn-seq study used a mariner-family transposon to generate random insertions across the UA159 genome (31). The transposon-genomic DNA junctions were amplified and sequenced to quantify fitness after growth in a defined medium (FMC). Genes lacking transposon insertions sites are predicted to be essential in FMC. Overall, 84.8% of the essentiality predictions from the iSMU model were consistent with the Tn-seq data (Fig. 4). A comparison between iSMU’s essential gene predictions and the Tn-seq data is shown in the iSMU map in Fig. 2.

The gene deletion strains in the work of Quivey et al. (32) were constructed individually using homologous recombination with a selective marker. The deletion library contains strains for 1,112 of the 1,956 genes in S. mutans UA159, including 366 of the 488 genes in the iSMU metabolic model. The remaining 122 genes are hypothesized to be essential or could not be deleted due to technical limitations. Deletion strains were grown in brain heart infusion medium (BHI), a rich and undefined medium. We simulated BHI by opening all exchange reactions in the iSMU model. As shown in Fig. 4, 78.1% of the experimental essentiality results agreed with the model predictions. Agreement between iSMU’s essential gene predictions and the data from the work of Quivey et al. (32) is highlighted in the iSMU map in Data Set S4.

### S. mutans has complex nutrient requirements.

Several studies have investigated the minimal requirements for S. mutans growth *in vitro* (3, 33–36). Like many obligate human pathogens, S. mutans requires a combination of carbon, nitrogen, sulfur, and phosphorus sources; inorganic minerals; nucleotides; and vitamins and cofactors. We used our iSMU model and phenotypic assays to systematically explore auxotrophies in S. mutans.

The S. mutans UA159 genome encodes complete biosynthetic pathways for all 20 amino acids (22). S. mutans can grow without any exogenous amino acids using ammonium as the sole nitrogen source (33). The iSMU model can similarly produce biomass with ammonium and no amino acids. Using a series of leave-one-out experiments, we confirmed that the removal of individual amino acids from a defined medium does not affect S. mutans growth *in vitro* (Fig. 3). Simultaneous removal of cysteine and cystine does not significantly reduce growth, indicating that S. mutans can catabolize another sulfur source, possibly sulfate or methionine.

S. mutans can theoretically synthesize nucleotides (adenine, guanine, cytosine, uracil, and thymine) but only through the nonoxidative branch of the pentose phosphate pathway (37). S. mutans UA159 apparently lacks the more efficient oxidative branch of the pentose phosphate pathway (38). The nonoxidative pentose phosphate pathway is bidirectional and can produce or recycle ribose 5-phosphate and other pentose sugars. These sugars are necessary precursors for nucleotide biosynthesis. The model iSMU requires no nucleotides in the medium for growth. However, we found that removing all nucleotides from CDM prevents growth of S. mutans (Fig. 3). We know that cytosine and thymine are not required for S. mutans growth since they are not present in CDM (Table 1). Consistent with model predictions, uracil and guanine can also be removed from CDM. Removing uracil does not significantly alter growth, but removing guanine causes a 30% decrease in growth rate (Fig. 3). Only the removal of adenine completely abolished growth in CDM, which does not agree with our model predictions (Fig. 3).

**TABLE 1:** CDM includes 22 amino acids, 11 vitamins, 3 nucleobases, 8 inorganic salts, and glucose

Component	Concn (g/liter)
Deionized H_2_O	
Iron	0.006
Phosphate	18.3
MgSO_4_·7H_2_O	0.7
MnSO_4_·H_2_O	0.005
NaC_2_H_3_O_2_·3H_2_O	4.5
dl-Alanine	0.1
l-Arginine	0.1
l-Aspartic acid	0.1
l-Asparagine	0.1
l-Cysteine HCl	0.65
l-Cystine	0.05
l-Glutamic acid	0.1
l-Glutamine	0.2
Glycine	0.1
l-Histidine	0.1
l-Isoleucine	0.1
l-Leucine	0.1
l-Lysine	0.1
l-Methionine	0.1
l-Phenylalanine	0.1
l-Proline	0.1
Hydroxy-l-proline	0.1
l-Serine	0.1
l-Threonine	0.2
l-Tryptophan	0.1
l-Tyrosine	0.1
l-Valine	0.1
*p*-Aminobenzoic acid	0.0002
Biotin	0.0002
Folic acid	0.0008
Nicotinamide	0.001
B-NADP	0.0025
Pantothenate Ca salt	0.002
Pyridoxal	0.001
Pyridoxamine diHCl	0.001
Riboflavin	0.002
Thiamine hydrochloride	0.001
Vitamin B_12_	0.0001
Adenine	0.02
Guanine hydrochloride	0.02
Uracil	0.02
CaCl_2_	0.00676
NaHCO_3_	2.5
Glucose	10.0

S. mutans is unable to synthesize thiamine, riboflavin, pyridoxal 5-phosphate, NAD^+^/NADP^+^, pantothenate, and folate. Anabolic pathways for these vitamins and cofactors are incomplete, and key enzymes are not encoded in the S. mutans UA159 genome (Fig. 2). All of these nutrients (or their metabolic precursors) are ingredients in two chemically defined media used to culture streptococci (CDM [39] and FMC [40]). Our model predicts that aminobenzoate (an ingredient in CDM) can substitute for folate, but at least one of these nutrients is required for growth. Indeed, we found that S. mutans UA159 can grow in CDM without either folate or aminobenzoate but is unable to grow in medium lacking both (Fig. 3).

S. mutans cannot synthesize NAD^+^/NADP^+^ *de novo*. The iSMU model predicts that both NAD^+^ and NADP^+^ can be produced from any of NAD^+^, NADP^+^, nicotinamide, or nicotinate alone. CDM includes two of these four metabolites (NADP^+^ and nicotinamide). Consistent with our model, S. mutans can grow in CDM missing either NADP^+^ or nicotinamide, but not both (Fig. 3).

### The iSMU model predicts carbon source utilization of S. mutans.

S. mutans can metabolize a wide range of carbon sources (41, 42), allowing the organism to thrive in the oral cavity of humans with varied diets. S. mutans’ robustness to changing carbon sources may be essential for its survival and cariogenicity. The iSMU model can grow in CDM with 24 carbon sources: glucose, fructose, sucrose, lactose, trehalose, ascorbate, arbutin, maltose, cellobiose, salicin, sorbitol, mannitol, mannose, *N*-acetylglucosamine, fructan, galactose, galactinol, epimelibiose, melibiitol, melibiose, raffinose, maltodextrin, stachyose, and malate. Growth on glucose, sucrose, and *N*-acetylglucosamine is consistent with previous experimental studies (41, 42). To validate our model, we tested iSMU’s growth predictions for 18 carbon sources (Table 2). The model predictions and experimental results agreed for 17 of the 18 carbon sources. The sole disagreement was growth on melibiose. The iSMU model can grow on melibiose, but S. mutans UA159 did not grow on this carbon source. Other S. mutans strains can catabolize melibiose (43–46). The iSMU model imports melibiose using a multiple sugar transporter (MsmEFGK) that reportedly supports melibiose catabolism under anaerobic conditions with a semidefined medium (47). It is possible that S. mutans UA159 requires an ingredient not found in CDM to utilize melibiose via MsmEFGK or that the strain’s melibiose pathway is incomplete or inactive.

**TABLE 2:** Model predictions and measured growth for S. mutans UA159 on 18 carbon sources

Carbon source	iSMU prediction	Expt
Glucose	Growth	Growth
d-Galactose	Growth	Growth
d-Trehalose	Growth	Growth
d-Mannose	Growth	Growth
d-Sorbitol	Growth	Growth
d-Mannitol	Growth	Growth
d-Fructose	Growth	Growth
Xylose	No growth	No growth
d-Lactose	Growth	Growth
Sucrose	Growth	Growth
d-Cellobiose	Growth	Growth
d-Raffinose	Growth	Growth
Salicin	Growth	Growth
Oxalic acid	No growth	No growth
Maltodextrin	Growth	Growth
Acetate	No growth	No growth
Melibiose	No growth	Growth
*N*-Acetylglucosamine	Growth	Growth

### iSMU predictions guide experiments.

**FIG 5:**
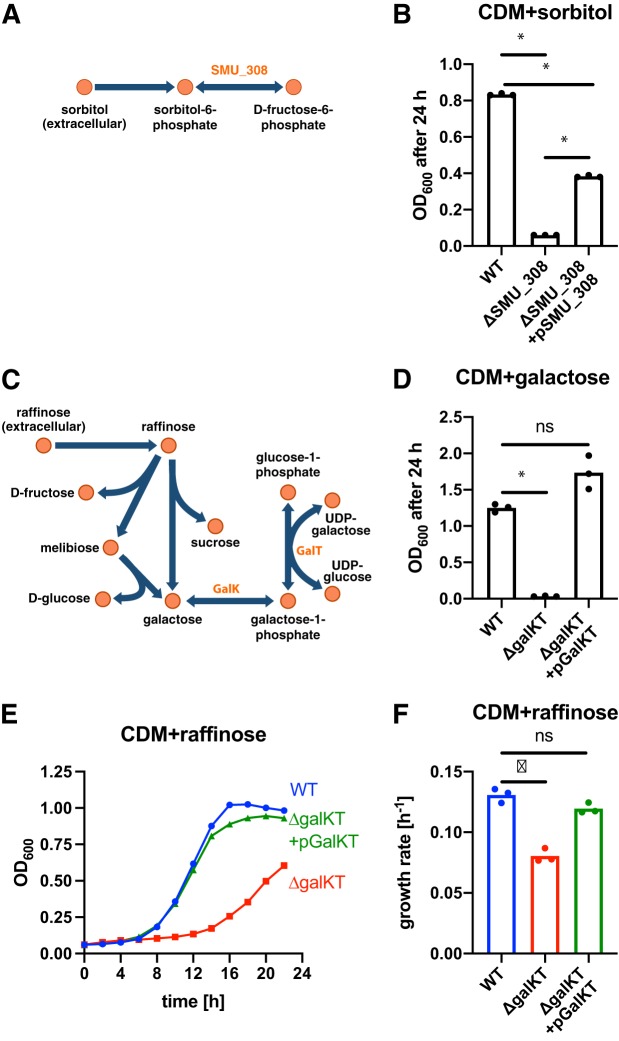
iSMU guides experiments with S. mutans. (A) The model predicts that sorbitol catabolism requires the product of SMU_308. (B) Wild-type (WT) S. mutans UA159 grows on sorbitol as a carbon source, but a strain with a scarless deletion of SMU_308 does not. Complementing the deletion strain with a plasmid carrying SMU_308 restores growth. (C) The Leloir pathway breaks down raffinose into galactose. Enzymes GalK and GalT are required to utilize galactose. There is no predicted exporter for galactose in S. mutans UA159. (D) The genes *galK* and *galT* are required for growth on galactose. (E) The Δ*galKT* strain has a growth defect on raffinose, potentially due to intracellular accumulation of galactose. (F) Adding the *galKT* genes back to the Δ*galKT* strain restores the growth rate on raffinose to wild-type levels. For panels B, D, and F, bar height indicates the mean from three biological replicates. * indicates *P < *0.001 by *t* test. ns, not significant. The representative growth curves in panel E correspond to the biological replicate in panel F with the median growth rate.

Metabolic models like iSMU link growth phenotypes to the organism’s genotype. These predictions can guide experiments to validate gene annotations for metabolic enzymes. The iSMU model predicts—and our experiments confirm—that S. mutans can grow on sorbitol (Table 2). There are several enzymatic pathways for sorbitol utilization, including conversion of sorbitol into fructose by a sorbitol dehydrogenase (EC 1.1.1.14 or 1.1.1.15), the conversion of sorbitol to sorbose (EC 1.1.99.21 or 1.1.1.289) and the subsequent utilization of sorbose, or the conversion of sorbitol into glucose by an aldehyde reductase (EC 1.1.1.21). In the iSMU model, sorbitol is phosphorylated and converted into fructose-6-phosphate by a putative sorbitol-6-phosphate 2-dehydrogenase encoded by the gene SMU_308 (Fig. 5A). If the model is correct, SMU_308 should be essential for growth on sorbitol.

We tested the predicted essentiality of SMU_308 by deleting the gene from S. mutans UA159. While the wild-type UA159 strain was able to grow on sorbitol, the ΔSMU_308 strain was unable to grow (Fig. 5B). Growth on sorbitol was restored when the SMU_308 gene was added back to the ΔSMU_308 strain on a plasmid (Fig. 5B). These experiments confirm the essentiality of gene SMU_308 for growth on sorbitol, supporting the model’s mechanism for sorbitol catabolism.

We used iSMU to predict more indirect links between genotype and phenotype. The model predicts that galactose is produced as a by-product during the utilization of oligosaccharides and sugar alcohols (raffinose, stachyose, epimelibiose, melibiose, galactinol, and melibiitol). These complex carbohydrates are broken down into simpler sugars like galactose that must be exported or catabolized by the cell. Thus, disrupting galactose catabolism can indirectly affect S. mutans’ ability to grow on oligosaccharides and sugar alcohols.

Flux balance analysis assumes a steady state with no net accumulation or depletion of metabolites. Our manual curation process for iSMU provided no genomic evidence for a galactose exporter, so intracellular galactose must be converted to another metabolite to avoid accumulation. Our simulations with iSMU agree—blocking galactose catabolism prevents growth on galactose or any complex carbohydrate that produces galactose as an intermediate metabolite.

Galactose utilization in S. mutans follows the Leloir pathway (Fig. 5C), including phosphorylation by galactokinase (GalK, EC 2.7.1.6) and subsequent conversion into UDP-galactose by a uridylyltransferase (GalT, EC 2.7.7.12) (48). Our model predicts that deletion of *galK* or *galT* (or both) would abolish galactose catabolism. We deleted both genes from S. mutans UA159 and confirmed that the Δ*galKT* strain is unable to grow on galactose (Fig. 5D). Adding the genes back to the Δ*galKT* strain via a plasmid is sufficient to restore growth on galactose (Fig. 5D). These results agree with previous mutagenesis studies of the Leloir pathway in S. mutans (49).

Our model predicts that the complex sugar raffinose is broken down into sucrose, fructose, and melibiose. The melibiose is further broken down into galactose and glucose. Growth on raffinose should indirectly depend on galactose catabolism to avoid a toxic buildup of galactose in the cell. Wild-type S. mutans UA159 grows on raffinose (Fig. 5E), but the Δ*galKT* mutant has a growth defect when raffinose is the sole carbon source (Fig. 5E and F). Adding the *galKT* genes back to the Δ*galKT* strain via a plasmid relieved the growth defect (Fig. 5E and F). These results demonstrate that the Leloir pathway is required for efficient growth on complex sugars like raffinose and suggest that accumulation of intermediate galactose may be toxic to the bacterium.

## DISCUSSION

iSMU is the first whole-genome metabolic model of the cariogenic pathogen S. mutans UA159. The model captures the entire metabolism of the organism and was validated by comparing model predictions to experimental evidence. Metabolism plays a dual role in the pathogenicity of S. mutans. First, fermenting sugars creates caries-causing lactic acid. A significant portion of the S. mutans genome is dedicated to carbohydrate metabolism, reflecting the plasticity of S. mutans’ metabolism. Second, multiple metabolic subsystems are required for S. mutans to tolerate acid and outcompete noncariogenic streptococci. A mathematical model allows us to investigate connections among metabolic pathways during pathogenesis.

iSMU’s predictions agree with most of the nutrient depletion experiments, but some of S. mutans’ auxotrophies are unexplained by the model. For example, the UA159 genome encodes a complete pathway for adenine synthesis, but exogenous adenine is required for growth *in vitro*. The adenine synthesis pathway in iSMU may not be expressed or functional in UA159 when grown aerobically in CDM. Other experiments agree qualitatively but not quantitatively with the model. When guanine, aminobenzoate, nicotinamide, or sodium acetate is removed from CDM, S. mutans grows slower, but that defect is not predicted by the model. The model also underpredicts growth rates when pyridoxal and pyridoxamine, riboflavin, and thiamine are removed. Differences like these are expected with constraint-based models that lack kinetic details for nutrient uptake and enzymatic turnover.

S. mutans UA159 can grow on minimal medium with ammonium as the sole nitrogen source (33), and the iSMU model can produce biomass under these conditions. Experimentally, growth on ammonium requires an anaerobic environment, but the model can produce biomass with or without oxygen. Oxygen may repress expression of enzymes required for scavenging nitrogen from ammonium, and the lack of regulation in our model would explain why iSMU can grow aerobically using ammonium.

Several factors could explain the disagreements between the model’s essentiality prediction and experimental results. First, we note that both methods for identifying essential genes have technical strengths and limitations. In the ordered gene deletion library, any gene for which a deletion mutant cannot be constructed is labeled essential. A nonessential gene located in a region of the chromosome refractory to homologous recombination would be incorrectly labeled as essential. The Tn-seq libraries in the work of Shields et al. (31) were constructed using *in vitro* transposon mutagenesis followed by homologous recombination, so the same limitation applies. The Tn-seq libraries were grown for ∼30 generations before sequencing. Such a large expansion can bias the library against mutants with large fitness defects. Although these mutants may be viable, they appear at such low frequency in the final pool that they are missed during sequencing. The corresponding genes would be incorrectly labeled as essential. Another factor that can explain the disagreement is that the wrongly predicted genes encode enzymes that may have secondary functions essential for the cell. A significant number of genes that were predicted to be nonessential encode kinases and phosphatases that may be involved in cell signaling. The disagreement between the Tn-seq and defined deletion library suggest that the “essential genome” of S. mutans UA159 has not been fully elucidated.

Inaccuracies in the iSMU model also contribute to disagreements over essential genes. After decades of curation, metabolic models for the model organisms E. coli and S. cerevisiae still miss some essential gene predictions (50, 51). Unannotated genes could catalyze redundant routes to synthesize essential metabolites *in vivo*, creating false-positive essentiality predictions in iSMU. Regulation, loss-of-function mutations, and missing cofactors can also restrict the metabolic capabilities of S. mutans, making the pathogen less metabolically flexible than the iSMU model.

Overall, we believe the model’s predictions could be improved by either (i) incorporating regulatory rules during simulations or (ii) using gene expression or other high-throughput data to tailor the model to anaerobic, aerobic, acidic, or other conditions. S. mutans’ metabolic requirements change as the bacterium encounters different niches in the mouth. Before the organism forms thick biofilms and deep dental caries, growth conditions are likely aerobic with abundant nutrients from saliva and food consumed by the host. Deep dental caries may create anaerobic conditions with limited nutrient availability. In this environment, S. mutans would need to synthesize many biomass components *de novo*.

The steady-state (or quasi-steady-state) assumption of FBA is often perceived as a limitation of the method. However, the steady-state assumption allows us to predict toxic accumulation of intermediates, as evidenced by our experiments with galactose and the Δ*galKT* strains. It is important to note that the buildup of galactose was due to the irreversibility of the galactose importer. If we assumed that the transporter was bidirectional, excess galactose could be exported and the model predictions would not have matched the organism’s phenotype. Careful curation of reversibility is just as important to model fidelity as correct genome annotations.

S. mutans is a model organism in oral microbiology (52). Our iSMU model draws from hundreds of studies to form an accurate, genome-wide picture of S. mutans metabolism. The model also highlights the value added by manual curation. The metabolism of S. mutans is well characterized on the molecular and pathway levels. Incorporating manually curated models of S. mutans and other lactic acid bacteria may improve the accuracy of automatic reconstruction pipelines.

## MATERIALS AND METHODS

### Model construction.

The metabolic network of S. mutans UA159 was reconstructed according to best practices in the COBRA modeling community (53). As summarized in Fig. 1A, reconstruction began with the annotated UA159 genome (RefSeq GCA_000007465.2). Metabolic enzymes and the associated reactions were initially collected from KEGG (54) and Uniprot (55). The Metacyc (56), RHEA (57), ModelSEED (25), BiGG (58), and ChEBI (59) databases were used as secondary sources for metabolic reactions. Transport reactions were verified with TransportDB (60). KEGG identifiers were used for metabolites and reactions for consistency with other databases. Reactions without gene associations were added only when supported by experimental or literature evidence. These 12 reactions are explained in Table S1 in the supplemental material. All chemical species and formulas were converted to their protonation state at pH 7.0 using the ModelSEED database. A custom map of the iSMU model was constructed using Escher version 1.6.0 (Fig. 2) (61).

10.1128/mSystems.00529-19.2 TABLE S1 GapFilled reactions in iSMU and evidence for inclusion. Download Table S1, PDF file, 0.05 MB. Copyright © 2019 Jijakli and Jensen. 2019 Jijakli and Jensen This content is distributed under the terms of the Creative Commons Attribution 4.0 International license.

### Model simulations using flux balance analysis.

All simulations were performed with Matlab (version R2016b; MathWorks, Natick, MA) using the COBRA toolbox (62). Mathematical programs were solved with Gurobi Optimizer (version 7.5; Gurobi Optimization, Beaverton, OR). Gene set enrichment for KEGG pathways was performed using KEGG Mapper (54). Details on simulations using flux balance analysis are available in Text S1 in the supplemental material.

10.1128/mSystems.00529-19.1 TEXT S1 Supplemental methods. Download Text S1, PDF file, 0.02 MB. Copyright © 2019 Jijakli and Jensen. 2019 Jijakli and Jensen This content is distributed under the terms of the Creative Commons Attribution 4.0 International license.

### Model availability.

The final model is available as an SBML file and a spreadsheet compatible with the COBRA toolbox (Data Sets S1 and S2). The model map is available as a JSON file (Data Set S3) and SVG image (Data Set S4). Future versions of the model and map will be available on the authors’ website (http://jensenlab.net).

10.1128/mSystems.00529-19.6 DATA SET S1 iSMU genome-scale metabolic model in SBML format. Download Data Set S1, TXT file, 1.1 MB. Copyright © 2019 Jijakli and Jensen. 2019 Jijakli and Jensen This content is distributed under the terms of the Creative Commons Attribution 4.0 International license.

10.1128/mSystems.00529-19.7 DATA SET S2 iSMU genome-scale metabolic model in spreadsheet format. Download Data Set S2, XLSX file, 0.08 MB. Copyright © 2019 Jijakli and Jensen. 2019 Jijakli and Jensen This content is distributed under the terms of the Creative Commons Attribution 4.0 International license.

10.1128/mSystems.00529-19.8 DATA SET S3 iSMU complete model map in JSON format. Download Data Set S3, TXT file, 1.2 MB. Copyright © 2019 Jijakli and Jensen. 2019 Jijakli and Jensen This content is distributed under the terms of the Creative Commons Attribution 4.0 International license.

10.1128/mSystems.00529-19.9 DATA SET S4 iSMU complete model map and essential genes from ordered array gene deletions. Download Data Set S4, PDF file, 1.3 MB. Copyright © 2019 Jijakli and Jensen. 2019 Jijakli and Jensen This content is distributed under the terms of the Creative Commons Attribution 4.0 International license.

10.1128/mSystems.00529-19.10 DATA SET S5 Gene essentiality predictions. Download Data Set S5, XLSX file, 0.03 MB. Copyright © 2019 Jijakli and Jensen. 2019 Jijakli and Jensen This content is distributed under the terms of the Creative Commons Attribution 4.0 International license.

### Strains and reagents.

Strains and plasmids are listed in Table 3. S. mutans UA159 (ATCC 700610) was cultured in Todd-Hewitt broth (63) with 0.3% yeast extract (THY) liquid medium or agar plates. Strains were grown overnight in 5% CO_2_ at 37°C unless specified. Growth experiments were performed in a chemically defined medium (CDM) (39) with 22 amino acids, 11 vitamins, 3 nucleobases, 8 inorganic salts, and a carbon source (Table 1). Two sets of growth experiments were performed: a set involving leave-one-out CDM variants with glucose as a carbon source and a set involving complete CDM with alternative carbon sources. All CDM-based media were prepared fresh weekly from concentrated stocks (39). All components were purchased from Sigma-Aldrich USA or Fisher Scientific USA and were sterilized by autoclaving or filtration.

**TABLE 3:** Bacterial strains and plasmids used in this study

Strain or plasmid	Description	Source or reference
Strains		
UA159	S. mutans type strain	ATCC 700610
UA159ΔSMU_308	Scarless knockout of sorbitol-6-phosphate 2-dehydrogenase (SMU_308); deleted bases 294120–297024	This study
UA159Δ*galKT*	Scarless knockout of galactokinase (*galK*, SMU_886) and galactose-1-phosphate uridylyltransferase (*galT*, SMU_887); deleted bases 838314–842938	This study
Plasmids		
pDL278	E. coli/streptococcal shuttle vector; Spec^r^	67
pSMU308	pDL278::SMU_308; SMU_308 includes the native promoter	This study
pGalKT	pDL278::*galKT*; *galKT* includes the native promoter	This study

Oligonucleotides were synthesized by Integrated DNA Technologies (Coralville, IA, USA). Peptides were purchased from GenScript, Inc. (Piscataway, NJ, USA). All enzymes were manufactured by New England Biolabs (Ipswich, MA, USA).

### Growth assays.

Overnight cultures of S. mutans were washed three times in sterile water or phosphate-buffered saline (PBS). The overnight culture was concentrated 5× (from 5 ml to 1 ml). For the leave-one-out experiments, 1 μl of the concentrate was used to inoculate wells of a 96 well plate containing 200 μl of defined medium. Plates were incubated without agitation in 5% CO_2_ at 37°C. Optical density was measured by absorbance at 600 nm every hour for 16 h using a Tecan Infinite 200 Pro plate reader (Tecan, Männedorf, Switzerland). Exponential growth rates were calculated using the R package CellGrowth (version 3.7; Ludwig Maximilian University of Munich [https://www.bioconductor.org/packages/release/bioc/html/cellGrowth.html]) with default settings. Growth rates were normalized to the growth rate in complete CDM.

For the carbon source growth experiments, 25 μl of the concentrated overnight culture was used to inoculate 5 ml of defined medium in culture tubes. The tubes were also incubated without agitation in 5% CO_2_ at 37°C. Optical density was measured by absorbance at 600 nm right after inoculation and at 24 h using a NanoDrop OneC (Thermo Fisher Scientific, Waltham, MA, USA).

### S. mutans transformation.

S. mutans was transformed by induced competence using a protocol adapted from reference 64. S. mutans UA159 was grown overnight in 5 ml THY or CDM plus glucose. The overnight culture was diluted 1:40 into 5 ml of fresh medium and grown to an optical density (OD) of ∼0.15. A 500-μl aliquot was placed in a 2-ml centrifuge tube, and 500 ng of competence-stimulating peptide (CSP-18; SGSLSTFFRLFNRSFTQA) was added. DNA (1 μg) was added after 20 to 30 min, followed by incubation for 2 h. Up to 250 μl of the mixture was plated on THY agar plates with 200 μg/ml spectinomycin (Sigma) or 500 μg/ml kanamycin (Sigma). Single colonies were picked after 24 to 36 h and grown in 5 ml THY under selection. For markerless mutants, the overnight culture was replated on THY agar plates with 4 mg/ml *p*-chlorophenylalanine (4-CP) (Sigma). All strains were verified by PCR amplification and Sanger sequencing (ACGT, Inc., Wheeling, IL).

### Markerless mutagenesis.

Gene deletion strains of S. mutans UA159 were constructed with direct repeat-mediated cloning-independent markerless mutagenesis (DR-CIMM) (65). DR-CIMM replaces the target gene with an antibiotic resistance marker (*aph3* conferring kanamycin resistance) and a negative selection marker (pheS*, a mutated phenylalanyl-tRNA synthetase conferring sensitivity to *p*-chlorophenylalanine [4-CP]). A small region of DNA directly upstream of the target gene is repeated downstream of pheS* to allow homologous recombination that removes both the *aph3* and pheS* genes. The target gene is deleted using selection on kanamycin. Subsequent plating on 4-CP selects for homologous recombination that removes both marker genes, leaving a scarless deletion.

The primer sequences in Table S2 in the supplemental material follow the DR-CIMM nomenclature in reference 65. Homology regions upstream and downstream of the target gene were amplified with primers upF, upR, dnF, and dnR. The direct repeat region was amplified with primers DR-F and DR-R. The *aph3* and pheS* cassette was amplified with primers IFDC3-F and IFDC3-R. All primers except upF and dnR contained tails to allow assembly by overlap extension PCR (66) into two constructs: (i) the upstream homology region plus the selection cassette and (ii) the selection cassette, direct repeat, and downstream homology region. S. mutans UA159 was transformed simultaneously with the two constructs.

10.1128/mSystems.00529-19.3 TABLE S2 Oligonucleotide sequences used in this study. Uppercase text represents overlap regions. Download Table S2, PDF file, 0.03 MB. Copyright © 2019 Jijakli and Jensen. 2019 Jijakli and Jensen This content is distributed under the terms of the Creative Commons Attribution 4.0 International license.

10.1128/mSystems.00529-19.4 TABLE S3 Curated genome-scale metabolic models used to compare number of reactions without a gene association and blocked reactions. Download Table S3, PDF file, 0.06 MB. Copyright © 2019 Jijakli and Jensen. 2019 Jijakli and Jensen This content is distributed under the terms of the Creative Commons Attribution 4.0 International license.

10.1128/mSystems.00529-19.5 TABLE S4 Model comparisons between iSMU, iML1515, and iBSU1144. Download Table S4, PDF file, 0.03 MB. Copyright © 2019 Jijakli and Jensen. 2019 Jijakli and Jensen This content is distributed under the terms of the Creative Commons Attribution 4.0 International license.

### Plasmid construction.

Genes *galKT* and SMU_308 were cloned into the shuttle vector pDL278 (67) to complement the deletion strains. The genes were amplified by Q5 polymerase using the primers in Table S2 containing tails with EcoRI restriction sites. Purified pDL278 was digested with EcoRI and dephosphorylated with rSAP. The linearized plasmid was ligated with the EcoRI-digested amplicon using T4 DNA ligase. The deletion strains were transformed using 5 μl of the ligation mixture.
