# Supplementary material for: Metabolic Modeling of Streptococcus mutans Reveals Complex Nutrient Requirements of an Oral Pathogen
Source: mSystems. 2019 Oct 29;4(5):e00529-19. doi: 10.1128/mSystems.00529-19 (PMC6819733; doi:10.1128/mSystems.00529-19)
Supplement: TABLE S1 [file mSystems.00529-19-st001.pdf]

Reaction	GPR	Enzyme	Evidence
R02735	SMU_Gap1	5.1.1.7	Leave one out CDM experiments show *S. mutans* can grow without lysine (see Figure 3)
R04620	SMU_Gap2	3.1.3.1	Leave one out CDM experiments show *S. mutans* can grow without folate if aminobenzoate is supplied (see Figure 3)
R02029	SMU_Gap3	3.1.3.27	*S. mutans* can produce cardiolipin (1)
R00238	SMU_Gap4	2.3.1.9	The reaction is necessary for the production of peptidoglycan. SMU_639, SMU_1392c, SMU_1654c, SMU_1730c, SMU_2055 and SMU_2072c encode putative uncharacterized acetyltransferases that can possibly catalyze this reaction
R00405	SMU_Gap5	6.2.1.5	Lysine and methionine biosynthesis requires succinyl-CoA. Leave one out CDM experiments show *S. mutans* can grow without lysine or methionine (see Figure 3).
R00529	SMU_Gap6	2.7.7.4	*S. mutans* can grow in a media with sulfate and thiosulfate as the only sulfur sources (2)
R00509	SMU_Gap7	2.7.1.25	*S. mutans* can grow in a media with sulfate and thiosulfate as the only sulfur sources (2)
R02021	SMU_Gap8	1.8.4.8	*S. mutans* can grow in a media with sulfate and thiosulfate as the only sulfur sources (2)
R00858	SMU_Gap9	1.8.1.2	*S. mutans* can grow in a media with sulfate and thiosulfate as the only sulfur sources (2)
R02239	SMU_Gap10	3.1.3.4	1,2-Diacyl-sn-glycerol is required to make cell wall components. There is genetic and enzymatic evidence that it can be recycled, but its biosynthesis is unclear.
R04858	SMU_Gap11	2.1.1.37	Recycles methionine into homocysteine. Only reaction missing in the pathway.
R00762	SMU_Gap12	3.1.3.11	Carbon source growth experiments show S. mutans can grow on fructose. PTS transporter transports fructose in D-Fructose 1,6-bisphosphate and this reaction converts the transported fructose into a required anabolic precursor.

1. MacGilvray ME, Lapek JD, Friedman AE, Quivey RG. 2012. Cardiolipin biosynthesis in Streptococcus mutans is regulated in response to external pH. Microbiology 158:2133–2143.

2. Martin EJS, Wittenberger CL. 1980. Regulation and function of ammonia-assimilating enzymes in Streptococcus mutans. Infect Immun 28:220–224.
