# Supplementary material for: Metabolic Modeling of Streptococcus mutans Reveals Complex Nutrient Requirements of an Oral Pathogen
Source: mSystems. 2019 Oct 29;4(5):e00529-19. doi: 10.1128/mSystems.00529-19 (PMC6819733; doi:10.1128/mSystems.00529-19)
Supplement: TEXT S1 [file mSystems.00529-19-s0001.pdf]

Model Simulation Framework

To simulate growth using iSMU, reactions were collected into a stoichiometric matrix S where element $S(i, j)$ corresponds to the stoichiometric coefficient of the model's i^{th} metabolite in the jth reaction. $S(i, j)$ is negative if the metabolite is consumed and positive if the metabolite is produced. Two vectors of lower (l) and upper (u) bounds determine the reversibility of reactions. A vector of reaction fluxes v was calculated by maximizing the flux through the biomass reactions subject to mass balance constraints ($Sv = 0$) and reversibility constraints ($l \leq v \leq u$). To simulate gene deletions, the gene/protein/reaction rules for each reaction were evaluated to identify reactions that cannot carry flux in the deletion strain. The upper and lower bounds of these reaction were set to zero before maximizing flux through the biomass reaction. Genes were considered essential if their deletion allowed no biomass flux.
