# Supplementary material for: Metabolic Modeling of Streptococcus mutans Reveals Complex Nutrient Requirements of an Oral Pathogen
Source: mSystems. 2019 Oct 29;4(5):e00529-19. doi: 10.1128/mSystems.00529-19 (PMC6819733; doi:10.1128/mSystems.00529-19)
Supplement: TABLE S2 [file mSystems.00529-19-st002.pdf]

Name	Description	Sequence
sorb_upF	SMU_308 upstream homologous region forward primer	cctgccatagcttttcgc
sorb_upR	SMU_308 upstream homologous region reverse primer with overlap with IFDC3 cassette	CTCTTTTTGTCATCAGAAAGCTTAAGTcctgaggaagctcctgttac
sorb_IFDC3-F	IFDC3 cassette forward primer with overlap with SMU_308 upstream homologous region	cagtaattgtaacaggagcttcctcaggACTTAAGCTTTCTGATGACAAAAAGAG
sorb_IFDC3-R	IFDC3 cassette reverse primer with overlap with SMU_308 direct repeat	ggcaaaataaaagccaagacaacagccttgGCCGGTCTCAAATTGCATG
sorb_DR-F	SMU_308 direct repeat forward primer with overlap with IFDC3	GTTTTAGGCATGCAATTTGAGACCGGCcaaggctgttgtcttggc
sorb_DR-R	SMU_308 direct repeat reverse primer with overlap with SMU_308 downstream homologous region	CCGCGCGTTTTACCTCCGGCAACAcctgaggaagctcctgttac
sorb_dnF	SMU_308 downstream homologous region forward primer with overlap with SMU_308's direct repeat	CAGTAATTGTAACAGGAGCTTCCTCAGGtgttgccggaggtaaaac
sorb_dnR	SMU_308 downstream homologous region reverse primer	ccatttcaaaggccaaggg
gal_upF	SMU_886 & SMU_887 upstream homologous region forward primer	caaactgacacgattggg
gal_upR	SMU_886 & SMU_887 upstream homologous region reverse primer with overlap with IFDC3 cassette	CTCTTTTTGTCATCAGAAAGCTTAAGTacgtgagtaaaggcttgg
gal_IFDC3-F	IFDC3 cassette forward primer with overlap with SMU_886 & SMU_887 upstream homologous region	gaattaaaccaagcctttactcacgtACTTAAGCTTTCTGATGACAAAAAGAG
gal_IFDC3-R	IFDC3 cassette reverse primer with overlap with SMU_887 & SMU_887 direct repeat	gagaaagaagggtgataatggctacGCCGGTCTCAAATTGCATG
gal_DR-F	SMU_886 & SMU_887 direct repeat forward primer with overlap with IFDC3	GTTTTAGGCATGCAATTTGAGACCGGCgtagccattatcacccttc
gal_DR-R	SMU_886 & SMU_887 direct repeat reverse primer with overlap with SMU_886 & SMU_887 downstream homologous region	CTCCAATACTCTCTACAAACCGCATGacgtgagtaaaggcttgg

gal_dnF	SMU_886 & SMU_887 downstream homologous region forward primer with overlap with SMU_886 & SMU_887 direct repeat	CAAGAATTAAACCAAGCCTTTACTCACGTcatgcggtttgtagagag
gal_dnR	SMU_886 & SMU_887 downstream homologous region reverse primer	gtatctggatctcccgaac
sorb_EcoRI_Fwd	Forward primer for the amplification of SMU_308 with the addition of EcoRI sites at the ends of the amplicon	taagcagaattccgaagaacttggtgctgag
sorb_EcoRI_Rev	Reverse primer for the amplification of SMU_308 with the addition of EcoRI sites at the ends of the amplicon	taagcagaattcttaaccgcgcgttttacc
gal_EcoRI_Fwd	Forward primer for the amplification of SMU_886 & SMU_887 with the addition of EcoRI sites at the ends of the amplicon	taagcagaattcatgtagccattatcacccttc
gal_EcoRI_Rev	Reverse primer for the amplification of SMU_886 & SMU_887 with the addition of EcoRI sites at the ends of the amplicon	taagcagaattctctgcacagtcgctagg
