# Supplementary material for: Metabolic Modeling of Streptococcus mutans Reveals Complex Nutrient Requirements of an Oral Pathogen
Source: mSystems. 2019 Oct 29;4(5):e00529-19. doi: 10.1128/mSystems.00529-19 (PMC6819733; doi:10.1128/mSystems.00529-19)
Supplement: TABLE S3 [file mSystems.00529-19-st003.pdf]

Model	Reactions	Blocked Reactions	Reactions without GPR	% Reactions without GPR	% Blocked Reactions
**AbyMBEL891**	891	324	891	100	36.4
**AraGEM**	1601	944	424	26.5	59
**GSMN. TB**	900	109	44	4.9	12.1
**PpaMBEL1254**	1254	575	1254	100	45.9
**PpuMBEL1071**	1071	397	1071	100	37.1
**STM v1. 0**	2546	947	511	20.1	37.2
**S. coilicolor**	1015	158	1015	100	15.6
**SpoMBEL1693**	1693	799	1693	100	47.2
**T. Maritima**	645	271	127	19.7	42
**VvuMBEL943**	943	301	943	100	31.9
**iAC560**	1112	398	449	40.4	35.8
**iAF1260**	2382	850	438	18.4	35.7
**iAF692**	690	207	181	26.2	30
**iAI549**	555	222	58	10.5	40
**iAN840m**	1129	548	131	11.6	48.5
**iAO358**	865	126	865	100	14.6
**iAbaylyiV4**	996	323	199	20	32.4
**iBT721**	778	120	250	32.1	15.4
**iBsu1103**	1681	349	244	14.5	20.8
**iCA1273**	2477	926	457	18.4	37.4
**iCB925**	938	437	64	6.8	46.6
**iCR744**	832	247	118	14.2	29.7
**iCS291**	493	49	493	100	9.9
**iCS400**	546	71	546	100	13
**iCac802**	1462	972	400	27.4	66.5
**iFF708**	1379	318	435	31.5	23.1
**iGB555**	633	140	633	100	22.1
**iHD666**	1373	541	1373	100	39.4
**iIB711**	971	65	207	21.3	6.7
**iIT341**	554	126	199	35.9	22.7
**iJL432**	560	215	164	29.3	38.4
**iJN678**	863	195	184	21.3	22.6
**iJN746**	1056	404	246	23.3	38.3
**iJO1366**	2583	878	460	17.8	34
**iJP815**	948	384	127	13.4	40.5
**iJR904**	1075	408	202	18.8	38
**iJS747**	705	336	88	12.5	47.7
**iKF1028**	959	310	959	100	32.3
**iLC915**	1423	420	405	28.5	29.5
**iLL672**	1195	86	1195	100	7.2
**iMA871**	1400	189	1400	100	13.5
**iMA945**	2300	473	2300	100	20.6
**iMB745**	825	246	196	23.8	29.8

**iMH551**	860	323	860	100	37.6
**iMM1415**	3725	1294	1515	40.7	34.7
**iMM904**	1577	692	534	33.9	43.9
**iMO1056**	1110	500	275	24.8	45
**iMP429**	556	125	132	23.7	22.5
**iND750**	1266	635	456	36	50.2
**iNJ661**	1025	285	305	29.8	27.8
**iNJ661m**	1049	234	325	31	22.3
**iNV213**	730	469	730	100	64.2
**iOG654**	784	199	784	100	25.4
**iOR363**	391	197	73	18.7	50.4
**iPP668**	1361	419	1361	100	30.8
**iPS189**	351	73	177	50.4	20.8
**iRC1080**	2191	610	419	19.1	27.8
**iRM588**	608	177	141	23.2	29.1
**iRR1083**	1285	226	267	20.8	17.6
**iRS1563**	1785	1286	344	19.3	72
**iRS1597**	1576	1208	383	24.3	76.6
**iRS605**	794	331	794	100	41.7
**iRsp1095**	1309	168	1309	100	12.8
**iSB619**	743	293	162	21.8	39.4
**iSH335**	374	85	374	100	22.7
**iSO783**	870	174	141	16.2	20
**iSR432**	581	230	581	100	39.6
**iSS724**	1616	583	1616	100	36.1
**iSS884**	1333	470	376	28.2	35.3
**iSyn669**	882	378	882	100	42.9
**iTH366**	1001	334	344	34.4	33.4
**iTL885**	1240	318	412	33.2	25.6
**iTY425**	719	136	719	100	18.9
**iVM679**	678	150	678	100	22.1
**iVS941**	774	272	774	100	35.1
**iWV1314**	2361	786	380	16.1	33.3
**iWZ663**	830	128	209	25.2	15.4
**iYL1228**	2262	1039	368	16.3	45.9
**iYO844**	1245	288	341	27.4	23.1
**iZM363**	880	253	880	100	28.8
**iZmobMBEL601**	601	199	601	100	33.1
**Mus musculus**	2037	1176	2037	100	57.7
**iBT721**	777	120	249	32	15.4
**iGB555**	633	140	633	100	22.1
**iMP429**	556	125	132	23.7	22.5
**iZM363**	880	409	880	100	46.5
**Average**				**53**	**32.7**
