# Supplementary material for: Metabolic Modeling of Streptococcus mutans Reveals Complex Nutrient Requirements of an Oral Pathogen
Source: mSystems. 2019 Oct 29;4(5):e00529-19. doi: 10.1128/mSystems.00529-19 (PMC6819733; doi:10.1128/mSystems.00529-19)
Supplement: TABLE S4 [file mSystems.00529-19-st004.pdf]

	iSMU	iML1515	iBSU1144
Reactions	675	2719	1083
Metabolites	429	1192	743
Genes in Model (% of total)	493 (24.2%)	1515 (35%)	719 (16.5%)
Total Genes	2040	4318	4352
Blocked Reactions (%)	17 (2.5%)	968 (35.6%)	735 (67.9%)
Biomass Components	56	101	63
