# Supplementary material for: Metabolic Modeling of Streptococcus mutans Reveals Complex Nutrient Requirements of an Oral Pathogen
Source: mSystems. 2019 Oct 29;4(5):e00529-19. doi: 10.1128/mSystems.00529-19 (PMC6819733; doi:10.1128/mSystems.00529-19)
Supplement: DATA SET S4 [file mSystems.00529-19-sd004.pdf]

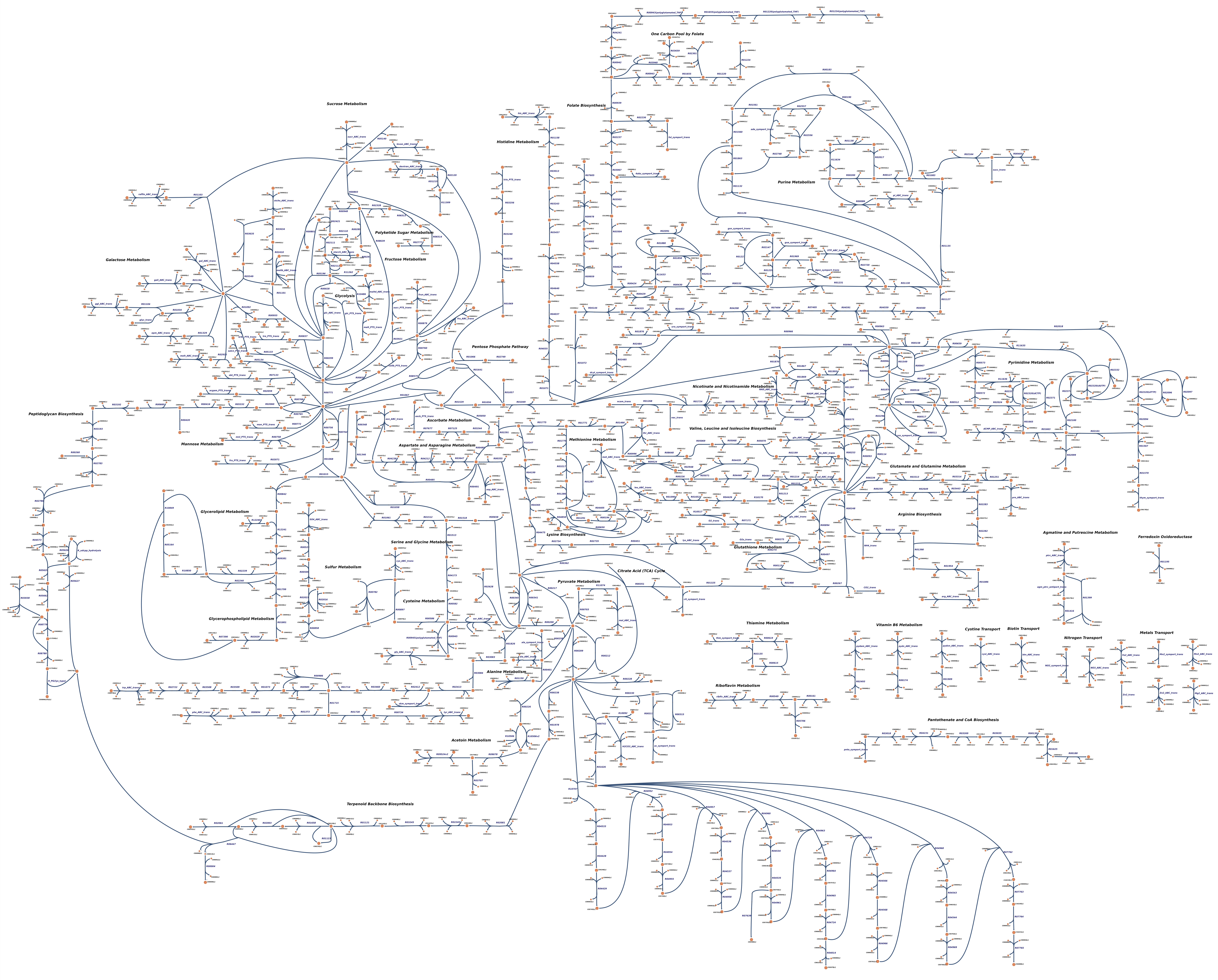
One Carbon Pool by Folate
Sucrose Metabolism
Folate Biosynthesis
Histidine Metabolism
Purine Metabolism
Galactose Metabolism
Polyketide Sugar Metabolism
Fructose Metabolism
Glycolysis
Pentose Phosphate Pathway
Pyrimidine Metabolism
Nicotinate and Nicotinamide Metabolism
Peptidoglycan Biosynthesis
Ascorbate Metabolism
Valine, Leucine and Isoleucine Biosynthesis
Mannose Metabolism
Methionine Metabolism
Aspartate and Asparagine Metabolism
Glutamate and Glutamine Metabolism
Glycerolipid Metabolism
Arginine Biosynthesis
Lysine Biosynthesis
Agmatine and Putrescine Metabolism
Ferredoxin Oxidoreductase
Serine and Glycine Metabolism
Glutathione Metabolism
Sulfur Metabolism
Citrate Acid (TCA) Cycle
Pyruvate Metabolism
Cysteine Metabolism
Glycerophospholipid Metabolism
Thiamine Metabolism
Vitamin B6 Metabolism
Cystine Transport
Biotin Transport
Metals Transport
Nitrogen Transport
Alanine Metabolism
Riboflavin Metabolism
Pantothenate and CoA Biosynthesis
Acetoin Metabolism
Terpenoid Backbone Biosynthesis

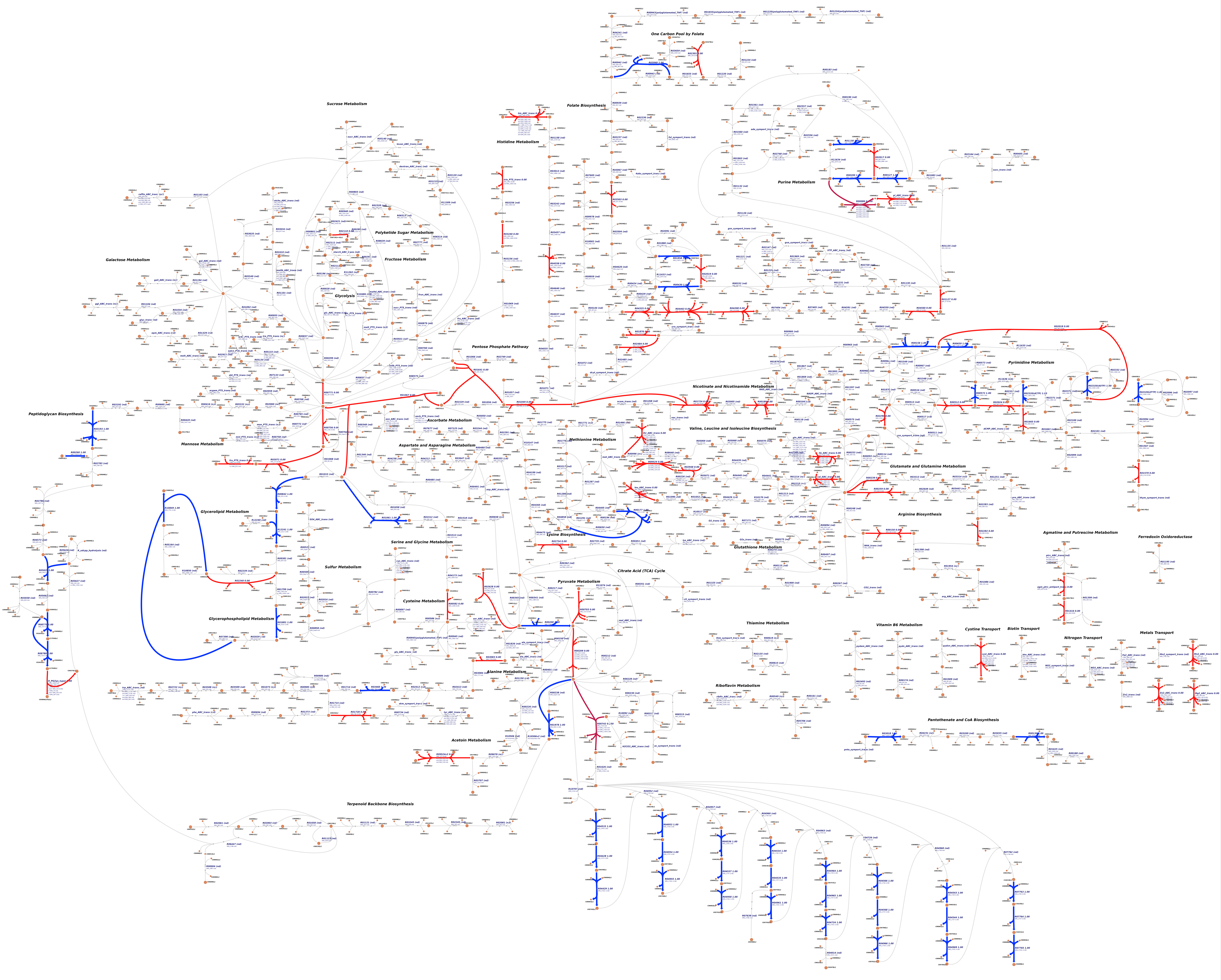
One Carbon Pool by Folate
Sucrose Metabolism
Folate Biosynthesis
Histidine Metabolism
Purine Metabolism
Polyketide Sugar Metabolism
Galactose Metabolism
Fructose Metabolism
Glycolysis
Pentose Phosphate Pathway
Pyrimidine Metabolism
Nicotinate and Nicotinamide Metabolism
Peptidoglycan Biosynthesis
Ascorbate Metabolism
Valine, Leucine and Isoleucine Biosynthesis
Mannose Metabolism
Methionine Metabolism
Aspartate and Asparagine Metabolism
Glutamate and Glutamine Metabolism
Glycerolipid Metabolism
Arginine Biosynthesis
Lysine Biosynthesis
Serine and Glycine Metabolism
Agmatine and Putrescine Metabolism
Ferredoxin Oxidoreductase
Glutathione Metabolism
Sulfur Metabolism
Citrate Acid (TCA) Cycle
Pyruvate Metabolism
Cysteine Metabolism
Glycerophospholipid Metabolism
Thiamine Metabolism
Vitamin B6 Metabolism
Cysteine Transport
Biotin Transport
Metals Transport
Nitrogen Transport
Alanine Metabolism
Riboflavin Metabolism
Pantothenate and CoA Biosynthesis
Acetoin Metabolism
Terpenoid Backbone Biosynthesis
